# Age- and sex-dependent susceptibility to phenobarbital-resistant neonatal seizures: role of chloride co-transporters

**DOI:** 10.3389/fncel.2015.00173

**Published:** 2015-05-12

**Authors:** Seok Kyu Kang, Geoffrey J. Markowitz, Shin Tae Kim, Michael V. Johnston, Shilpa D. Kadam

**Affiliations:** ^1^Neuroscience Laboratory, Hugo Moser Research Institute at Kennedy KriegerBaltimore, MD, USA; ^2^Department of Neurology, Johns Hopkins University School of MedicineBaltimore, MD, USA; ^3^Department of Pediatrics, Johns Hopkins University School of MedicineBaltimore, MD, USA

**Keywords:** neonatal seizures, ischemia, KCC2, NKCC1, phenobarbital, bumetanide

## Abstract

Ischemia in the immature brain is an important cause of neonatal seizures. Temporal evolution of acquired neonatal seizures and their response to anticonvulsants are of great interest, given the unreliability of the clinical correlates and poor efficacy of first-line anti-seizure drugs. The expression and function of the electroneutral chloride co-transporters KCC2 and NKCC1 influence the anti-seizure efficacy of GABA_A_-agonists. To investigate ischemia-induced seizure susceptibility and efficacy of the GABA_A_-agonist phenobarbital (PB), with NKCC1 antagonist bumetanide (BTN) as an adjunct treatment, we utilized permanent unilateral carotid-ligation to produce acute ischemic-seizures in post-natal day 7, 10, and 12 CD1 mice. Immediate post-ligation video-electroencephalograms (EEGs) quantitatively evaluated baseline and post-treatment seizure burdens. Brains were examined for stroke-injury and western blot analyses to evaluate the expression of KCC2 and NKCC1. Severity of acute ischemic seizures post-ligation was highest at P7. PB was an efficacious anti-seizure agent at P10 and P12, but not at P7. BTN failed as an adjunct, at all ages tested and significantly blunted PB-efficacy at P10. Significant acute post-ischemic downregulation of KCC2 was detected at all ages. At P7, males displayed higher age-dependent seizure susceptibility, associated with a significant developmental lag in their KCC2 expression. This study established a novel neonatal mouse model of PB-resistant seizures that demonstrates age/sex-dependent susceptibility. The age-dependent profile of KCC2 expression and its post-insult downregulation may underlie the PB-resistance reported in this model. Blocking NKCC1 with low-dose BTN following PB treatment failed to improve PB-efficacy.

## Introduction

Neonatal seizures are the most frequent clinical manifestation of central nervous system dysfunction in newborns, with an incidence of 1.5–3.5/1000 in term newborns, and an incidence as high as 10–130/1000 in preterm newborns (Evans and Levene, 1998; Volpe, 2001; Clancy, 2006; Tekgul et al., 2006; Bassan et al., 2008). Ischemia is a major cause of neonatal seizures (Lynch et al., 2002) and first-line anticonvulsant pharmacotherapy by commonly used anti-seizure drugs like PB often proves insufficient (Hunt and Inder, 2005; Kossoff, 2011). Both animal-model and human studies suggest that neonatal seizures themselves may worsen brain injury, decrease the threshold for subsequent seizures, and result in poor long-term neurological co-morbidities (Lombroso, 1996, 2007; Wirrell et al., 2001; Holmes, 2002; Clancy, 2006). Electroclinical dissociation is now a fairly well-accepted concept in human neonates, wherein GABA agonists are able to block the clinical manifestations of seizures; however, as displayed with electrographic monitoring, the brain continues to seize (Weiner et al., 1991; Scher, 2003; Staley, 2006; Evans et al., 2007; Rakhade and Jensen, 2009).

Early in development, the depolarizing GABA_A_ergic signaling that is instrumental in normal neuronal differentiation and migration has been shown to be responsible for the inefficacy of GABA_A_ agonists like PB, as an anti-seizure agent (Dzhala et al., 2008, 2010; Kahle et al., 2008). Therefore, cation chloride co-transporters, specifically NKCC1 and KCC2, could be used as potential targets for novel anti-seizure and anti-epileptogenic treatments (Loscher et al., 2013). NKCC1 is expressed in neurons and astrocytes throughout the brain, systemically in the kidney and inner hair cells of the ear (Lytle et al., 1995; Delpire et al., 1999; Flagella et al., 1999), and is robustly involved in early neural development (Pfeffer et al., 2009). The co-transporter KCC2 is CNS-specific predominantly in neurons and has a developmental expression profile that increases exponentially during the third trimester and continues to increase post-natally with advancing age (Dzhala et al., 2005; Kaila et al., 2014).

BTN, a potent NKCC1 antagonist that has been used as a diuretic in newborns, was also shown to be effective in reducing kainic acid-induced and hypoxic seizures in neonatal animals by blocking NKCC1 (Dzhala et al., 2008; Cleary et al., 2013) especially when used in combination with PB (Dzhala et al., 2008). BTN later became the focus of clinical trials to test its efficacy in seizing neonates with hypoxic ischemic encephalopathy (HIE; BTN clinical trial in Europe, 2009^1^; BTN clinical trial in USA, 2009^2^). However in pre-clinical studies, the anti-seizure effect of BTN has been shown to depend on the experimental model. BTN enhanced, suppressed, or had no effect on paroxysmal activity *in vitro* in different models, none of which modeled ischemia (Kilb et al., 2007; Vanhatalo et al., 2009; Kang and Kadam, 2014; Puskarjov et al., 2014b). Additionally, a recent study showed that kainic acid-induced seizures increased the surface expression of KCC2 (Khirug et al., 2010), shifting E_(GABA)_ close to the adult levels. However, under ischemic conditions, a substantial neuron-specific downregulation of KCC2 expression has been reported (Rivera, 2004; Jaenisch et al., 2010), which was also detected in our mouse model of neonatal ischemia. Such discordant model-specificity in the post-injury KCC2 expression may significantly alter the efficacy of drugs that depend on the Cl^−^ gradient for their anti-seizure effects. In new developments, the European clinical trial has recently been terminated for non-efficacy of BTN with associated ototoxicity following HIE induced seizures in neonates (Pressler et al., 2015).

This study utilized cerebral ischemia alone to induce acute ischemic seizures in the CD1 mouse strain, and investigated the following: (1) Quantitative analyses of EEG recordings of early post-stroke events at P7, P10, and P12 to determine the acute age-dependent seizure susceptibilities and seizure burdens following ischemia. (2) Response of the ischemic seizures to standard anti-seizure agent PB, as well as novel agent BTN at doses similar to the clinical trials, to evaluate for the first time, the effect of the NKCC1 blocker BTN, as an adjunct in a model of acute ischemic seizures. (3) Effect of neonatal ischemia on the developmental expression profile of KCC2 and NKCC1.

## Methods

### Experimental design

**Figure 1:**
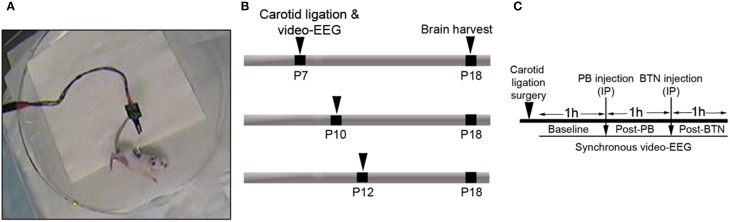
**(A)** Representative video-frame from a ligated pup having an ischemic tonic-clonic seizure. **(B,C)** Schematics of the time-line and experimental design.

This study was carried out in strict accordance with the recommendations in the Guide for the Care and Use of Laboratory Animals of the National Institutes of Health. The protocol was approved by the Committee on the Ethics of Animal Experiments of the Johns Hopkins University (Permit Number: A3272-01). All surgery was performed under isoflurane general anesthesia, and all efforts were made to minimize suffering. All litters of CD1 mice were purchased from Charles River Laboratories Inc. (Wilmington, MA). Newly born litters of pups arrived at post-natal 3 or 4 days old, and were allowed to acclimate. Animals were housed in polycarbonate cages on a 12 h light-dark cycle and food provided *ad libitum*. Ninety two pups from 12 litters were included in the video-EEG study (*n* = 48 male and *n* = 44 female). The susceptibility to ischemic seizures was tested at 3 developmental ages i.e., 7, 10, and 12 days old CD1 pups (*n* = 80 ligated and 12 shams). The experimental paradigm is depicted in Figure 1. The pups underwent permanent ligation and sham surgeries followed by 3 h of EEG recording each, as described in methods (total pups = 92 of which ligates P7 = 29, P10 = 24, and P12 = 27 and sham P7 = 5, P10 = 4, and P12 = 3; see Supplementary Table 1). From the ligated group of pups (total *n* = 80), the ligated-control pups also underwent 3 h EEG recordings to evaluate natural progression of ischemic seizure burden over the duration of treatment efficacy evaluated in this study (total pups *n*/*n* = 26/80, P7 = 8, P10 = 9, and P12 = 9). Following ligation, the ligated-treated group of pups [total pups *n*/*n* = 52/80; P7 = 20 (13 male and 7 female); P10 = 16 (8 male and 8 female), and P12 = 18 (9 male and 9 female)] were used to evaluate every baseline EEG (i.e., 1st h of recording) that was then compared to post-PB (2nd h of recording) and post-BTN (3rd h of recording) EEGs. Since the severity of stroke-injury and seizure varies between pups, the baseline EEG of each pup served as an important internal control. Additionally, since the exact time-point of onset for ischemia in human neonates is not known and may not be a single massive event, the 1 h of non-treatment also served as an important delay expected in the treatment of neonatal seizures that are detected hours after stroke in humans. There was an age-dependent mortality associated with survival to age P18 following the ischemic seizures in this study [i.e., 8 out of 29 pups at P7 (2 males and 6 females); 6 out of 24 pups at P10 (3 males and 3 females); and none at P12].

### Surgical procedure for ischemic insult and electrode implantation

Animals were anesthetized with 2% isoflurane. The right common carotid artery was permanently double-ligated with 6-0 surgisilk and the outer skin closed with 6-0 monofilament nylon to induce ischemia (i.e., ischemia was induced by unilateral common carotid-ligation alone. Unlike other rat models commonly used for neonatal stroke, no global hypoxia is needed to induce acute ischemic seizures). Since the ligated carotid is not transected in our model, the intact pulsating carotid artery with silk ligatures can lead to reperfusion over time in this model. The constrictive efficacy (which drops cerebral perfusion to 40% or less) of the silk ligatures is known to diminish, due to the loss of their tensile strength *in-vivo* against the pulsating artery as a function of time (Kadam et al., 2008a). Sham-control animals were treated identically except for the carotid ligation, and did not seize (Supplementary Figure 2A). The animals were then implanted with three sub-dermal EEG electrodes (1 recording, 1 reference, and 1 ground) on the skull overlying the parietal cortex using bregma as a reference. Scalp wire electrodes made for sub-dermal use in humans (IVES EEG; Model # SWE-L25—MA, USA) were implanted, sub-dermally fixed with adhesive, in the pups. The suggested spacing for human scalp electrodes is 5–8 mm, which allows for optimal acquisition of EEG signal from mouse brains (Freeman et al., 2003). In this study, the recording and reference electrodes were implanted less than 1 cm apart. Pups were then allowed to recover from anesthesia in a 36°C isothermal chamber for 3 h recording of video-EEG (Figure 1). At the end of the recording session, the pups were returned to the dams after removal of the sub-dermal electrodes and application of local anesthetic medication.

### *in vivo* synchronous video-EEG recording

After the completion of the surgical procedures (i.e., ligation + electrode implantation; 8 min + 8 min ~ 16 min total), the EEG recordings were initiated after the pups recovered from anesthesia. Bumetanide [0.1–0.2 mg/kg dissolved in 100% alcohol, aliquoted, and stored at −20°C; protocol similar to previous study (Dzhala et al., 2005)], phenobarbital (25 mg/kg dissolved in 0.9% sodium chloride, made on the day of experiment), or 0.9% sodium chloride was injected intraperitoneally (IP) after baseline post-stroke EEGs were recorded (Figure 1C). Since these experiments were also designed to test BTN-efficacy as an adjunct therapy to PB (based on the currently recruiting clinical trials), ligated mice underwent one of two regimens: (1) Treatment with saline only at 1 h and 2 h post-surgery; (2) Treatment with PB at 1 h and BTN at 2 h post-surgery. The treatment regimen of BTN before PB or BTN without PB was not relevant to this translational design. Pharmacokinetics of PB and BTN additionally supports our treatment regimen, because PB is a long acting drug even in immature rodents [half-life ~15 h (Markowitz et al., 2010) whereas BTN has a very short half-life of <30 min (Cleary et al., 2013)]. Data acquisition was done using PAL-8400 software with synchronous video capture (Pinnacle Technology Inc.). The data acquisition and conditioning system had a 14-bit resolution, sampling rates of 500 Hz, high pass filters of 0.5 Hz, and low pass filters of 1 kHz. The files were stored in.EDF format and scored in real time using the review and scoring software package. Manual scoring of all EEG files was done blinded to treatment protocols by simultaneously scoring EEG traces and the synchronous video in real time. Seizure burden scoring was done on EEG with sampling rates of 400 Hz that had a pre-amplifier gain of 100. The filters of 1 Hz high-pass and 60 Hz low-pass were used to remove ambient noise, and the binning was done in 10 s intervals. Seizures were defined as electrographic ictal events that consisted of rhythmic spikes of high amplitude, diffuse peak frequency of ≥7-8 Hz (i.e., peak frequency detected by automated spectral power analysis) lasting more than 6 s. Short duration burst activity lasting <6 s (brief runs of epileptiform discharge) was not included for seizure burden calculations in this study (Supplementary Figure 1).

### Behavioral seizure scoring after EEG seizure detection

**Figure 2:**
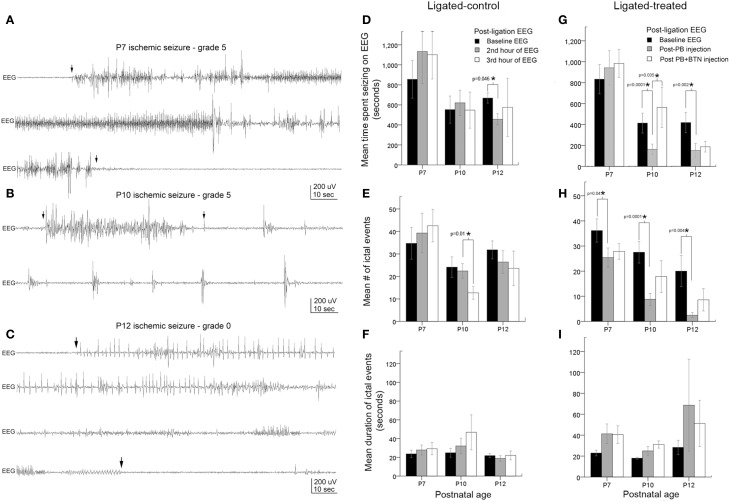
**Age-dependent seizure burden and PB-efficacy. (A–C)** Representative electrographic traces of ischemic seizures recorded with sub-dermal scalp electrodes at P7, P10, and P12 and their associated behavioral grades on video. Arrowheads show the start and end of ictal events. **(D–F)** EEG seizure burden, mean number of ictal events, and ictal durations in ligated-controls that received saline injections at each hour after ligation. Seizure burden after ischemia at baseline recording was highest at P7, and was significantly more severe than at P10 (*p* = 0.01) and P12 (*p* = 0.03): pairwise *t*-test **(G–I)**. Electrographic seizure burden, mean number of ictal events, and ictal durations in ligated-treated mice that received PB (1 h post-ligation) and BTN (2 h post-ligation; adjunct to PB). PB (25 mg/kg; IP) was inefficacious as an anti-seizure agent at P7. At the same loading dose, PB was significantly efficacious as an anti-seizure agent at P10 and P12. BTN as an adjunct failed to improve PB-efficacy at any age tested, and significantly blunted PB-efficacy at P10. PB-efficacy at P10 and P12 **(G)** was due to significant reduction in mean number of ictal events **(H)**. Ictal durations were not significantly different at any age tested (**I**).

After an EEG seizure was detected, its time and duration of occurrence were noted, and the synchronous behavioral activity recorded on video was scored according to a seizure rating scale modified for mouse pups from a previously reported scale used for adult mice (Morrison et al., 1996; Kadam et al., 2008b). Behavioral seizures recorded on video for EEG-identified seizures (Figures 2A–C; Supplementary Figure 1) were scored as follows: 0 = motionless/inactive; 1 = flexor spasms; 2 = jittery movements; 3 = repetitive grooming/scratching, circling toward side of ischemia, with head bobbing (Supplementary Video 1); 4 = limb clonus, unstable posture; 5 = mice that exhibited level four behaviors for >30 s or with loss of posture (Supplementary Video 2); and 6 = severe tonic-clonic behavior with inability to regain loss of posture. After video-EEG recording, the mice were returned to the dam and littermates. Electrographic seizures associated with grade 0–2 behaviors were grouped as non-convulsive seizures and grade 3–6 behaviors were graded as convulsive seizures.

### Histology

All animals were anesthetized with chloral hydrate (90 mg/ml; IP) before being transcardially perfused with saline and 10% formalin in phosphate buffer (pH 7.4). The whole brain was removed and submerged in the same fixative. The brains were cryoprotected by first immersing in 15% sucrose for 24 h, followed by 30% sucrose for 24 h. The brains were rapidly frozen using dry ice and placed in −80°C storage. Coronal brain sections (40 μm thick) were cut on a cryostat in serial order to create 5 series of sections and mounted on super frost plus glass slides. One series of sections from the EEG recorded pups was cresyl violet (CV) stained to quantitate ischemia injury using a previously described method of computer-assisted comparison of brain tissue area (Kadam et al., 2008b) in ipsilateral vs. contralateral hemispheres of fixed CV stained mouse brain sections (i.e., Basic MCID). Stroke injury severities were quantitated at P18 for all brains processed for CV (*n* = 49/80; P7 = 15, P10 = 15, and P12 = 19) to compare differences in infarct injury evolution between age groups. The remaining brains from study were fresh frozen for western blot analysis.

Immunohistochemistry (IHC) with triple labeling: Serial sections from frontal and parietal cortex and dorsal hippocampi from a separate cohort of naïve CD1 pups aged P3 to P22 were labeled with neuronal marker NeuN (1:100, Chemicon International; Catalog # MAB377) and then processed for double labeling with NKCC1 [1:100, Chemicon International: detecting 22 amino acid peptide sequence near the C-terminus (exon 21); Catalog # AB3560P] and KCC2 (1:200, Upstate: targeting N-terminal His-tag fusion protein corresponding to residues 932–1043; Catalog # 07-432). The NKCC1 antibody used in this study is similar to those used in related published literature and is incapable of detecting NKCC1b, due to the post-transcriptional splicing of exon 21 that overlaps with the targeted epitope site for the antibody (Plotkin, 1997; Dzhala et al., 2005; Aronica, 2007). Currently no pan-NKCC1 (i.e., splice isoforms a and b) antibodies are available. To block nonspecific binding, sections were first incubated for 1 h at room temperature (RT) in a solution containing 0.2% Triton X-100 and 10% normal goat serum in PBS (Invitrogen). The sections were incubated with primary antibodies overnight at 4°C. After 3 × 10 min wash in PBS, slides were incubated at RT with secondary antibodies (Alexa flour 488 and 594). After secondary antibody incubation, sections were washed 3 × 10 min in PBS and cover-slipped with an anti-fade medium for further image processing (Axiovision, Zeiss). Confocal microscopy of FV 1000 system with Olympus IX81 inverted microscope stand was used to acquire images of immuno-stained sections. Z-stack images with 5 um step-size for bilateral cortices was obtained from coronal sections cut at 40 μm thickness. The Z-stacks were fused to obtain the final images.

### Western blots

All animals for immunochemical characterizations were anesthetized with chloral hydrate (90 mg/ml; IP) before being transcardially perfused with ice-cold saline. The whole fresh brains were removed, separated into left and right cerebral hemispheres and frozen in liquid nitrogen and stored at −80°C in preparation for further processing. Brain tissue homogenates were made and suspended in RIPA buffer containing one Complete Mini, Ethylenediaminetetraacetic acid (EDTA)-free protease inhibitor cocktail tablet (Roche Indianapolis; Catalog # 04693159001) per 10 mL of buffer. Total protein amounts were measured using the Bradford protein assay (Bio-Rad) and samples diluted for equal amounts of protein in each sample. Fifty micrograms of protein samples were run on 4–12% gradient SDS gels (Bio-Rad) and transferred onto polyvinylidene difluoride (PVDF) membranes. Membranes were blocked for 1 h in odyssey buffer before overnight incubation in primary antibodies, NKCC1 and KCC2. Blots were then incubated for 1 h in secondary antibodies (Licor; IR Dye 800CW Donkey anti-Rabbit IgG: Product # 926-32213 and IR Dye 680LT Goat anti-Mouse IgG: Product # 926-68070). Protein bands were visualized by chemiluminescence, using the Odyssey infrared imaging system 2.1 (LI-COR biosciences). The optical density of each sample was normalized to the level of expression of the actin run on each blot, for each antibody for statistical analysis. KCC2 and NKCC1 expression profiles in ipsilateral ischemic hemispheres were normalized to contralateral non-ischemic hemispheres in the same brain homogenized samples to compare differential effects of ischemic injury in the model.

### Statistics

Group means for seizure severity between treated and control mice were analyzed with independent sample student's *t*-tests. Treatment efficacy following PB+BTN administration for seizure burdens was analyzed using repeated measures One-Way ANOVAs followed by pairwise *t*-tests. Mauchly's test was used to confirm the equal variances of the differences detected in repeated measures ANOVA, which is known for testing a statistical assumption of sphericity. No significance detected in Mauchly's test indicates that the assumption of sphericity for the relevant *F*-test in repeated measure ANOVA is not violated (Mauchly, 1940). Non-parametric correlations among severities of infarct lesion, seizure counts, and seizure frequency (both electrographic and behavioral) were assessed by Spearman's test. Differences with *p* < 0.05 were considered statistically significant.

## Results

### Age-dependent susceptibility to ischemic seizures and response to anticonvulsants

EEG recordings in the post-ligation period (Figure 2) revealed ictal events along with interictal spikes and non-convulsive epileptiform discharges between ictal events (i.e., short duration bursts lasting 2–5 s). Ictal events on EEG (i.e., lasting >6 s) associated with video correlates where pup was seen to be motionless or inactive (grade 0) or ictal events with behavioral correlates of grade 1 or 2 (i.e., scale 1–6; Morrison et al., 1996) were graded as non-convulsive (since these behaviors are also seen in naïve littermates), as described in methods. The baseline acute ischemic seizures recorded in the 1st post-ischemic hour were significantly more severe at P7 (ligated control and ligation-treated group pooled) compared to P10 and P12 (Figures 2D,G, black bars, *p* < 0.0001 between P7 vs. P10 and P7 vs. P12). The ligated-control group of P10 pups showed a significant decrease in the number of ictal events in the 3rd h of EEG recording (Figure 2E) not seen at P12.

Ligated-treated pups using the same 3 h recording paradigm with PB (25 mg/kg) treatment at 1 h post-ligation and BTN (0.1–0.2 mg/kg) as an adjunct 2 h post-ligation followed similar trends for baseline EEG severities as the ligated-control group (Figures 2G–I, black bars, Supplementary Figure 3). However, interesting age-dependent drug efficacies were detected within the treated group of pups. At P7, PB and BTN both failed to have any significant anti-seizure effect on total time spent seizing on EEG (Figure 2G, Supplementary Figure 3). PB, however, did modulate the ischemic seizures by significantly reducing the number of ictal events in the 2nd h recording (Figure 2H, *p* = 0.04). The lack of overall PB-efficacy as an anti-seizure therapy resulted from a sustained increase in the duration of post-treatment ictal events (Figure 2I) that was not seen in the ligated-control group (Figure 2F). Adjunct BTN administration did not improve PB-efficacy, and the longer ictal durations persisted at P7. In contrast, at P10, PB administration 1 h after ligation, significantly reduced the seizure burden by 61% (Figure 2G, gray bar; Supplementary Figure 3) by significantly reducing the number of ictal events (Figure 2H, gray bar). No significant effect on the duration of the ictal events was detected (Figure 2I, gray bar). Follow-on BTN administration, failed to improve PB-efficacy, and additionally PB-induced seizure suppression was lost at P10 (Figure 2G; Supplementary Figures 3, 6). This significant aggravation of seizures was driven by an increase in the number of ictal events (Figure 2H; Supplementary Figure 6) following BTN IP injection. At P12, post-PB seizure suppressions were similarly effective compared to P10. PB treatment after 1st h significantly reduced the seizure burden by 64% due to a significant reduction in overall number of ictal events. BTN again failed to act as an effective adjuvant as it did not improve PB-efficacy (Figure 2G; Supplementary Figure 6).

When ligated-control and ligated-treated groups were directly compared to each other by pairwise *t*-tests, no significant difference was detected among the different groups for baseline seizure burden at P7 (*p* = 0.67, black bars Figure 2D compared to Figure 2G), P10 (*p* = 0.9, black bars Figure 2D compared to Figure 2G), or at P12 (*p* = 0.1, black bars Figure 2D compared to Figure 2G). Additionally, at P7, post-treatment seizure burdens in ligated-treated group were not significantly different from the ligated-control group in the 2nd and 3rd h of recordings [*P* = 0.6 (gray bars) and 0.6 (white bars), respectively]. In contrast, at P10 and P12, post-PB (i.e., 2nd h) seizure burden was significantly lower in ligated-treated group than in the ligated-control group during the same period (*p* = 0.007 and 0.03, respectively, gray bars).

Repeated measures ANOVAs using a within subjects design for efficacious drug effects using the PB+BTN protocol in the ligation-treated group were also evaluated. At P7, Mauchly's test for sphericity was not significant (*p* = 0.2) and within-subject drug effect was not significant (*df* = 2, *F* = 0.62, and *p* = 0.55). At P10, Mauchly's test for sphericity was significant (*p* = 0.02) and within-subjects drug effect was also significant (*df* = 2, *F* = 6.54, and *p* = 0.01). The within-subjects contrast showed that the linear drug effect was not significant, but the quadratic drug effect was significant (*p* = 0.01). At P12, Mauchly's test for sphericity was not significant (*p* = 0.9) and within subject drug effect was not significant (*df* = 2, *F* = 3.690, and *p* = 0.06). At P12, the within-subjects contrast was not significant for either the linear or quadratic drug effect. In summary, PB was a significantly efficacious anti-seizure agent both at P10 and P12 when evaluated by within-group pairwise *t*-tests and by independent sample *t*-tests compared to the ligated-control group. However at P7, PB+BTN failed to act as an efficacious anti-seizure therapy. At all three ages evaluated, BTN failed to improve PB-efficacy, but significantly blunted the PB-subdued ischemic seizures at P10. The overall lack of BTN-efficacy in the model and PB inefficacy at P7 could not be attributed to the clustering of seizures (see Supplementary Figure 6).

### Non-convulsive vs. convulsive seizures at P7, P10, and P12

**Figure 3:**
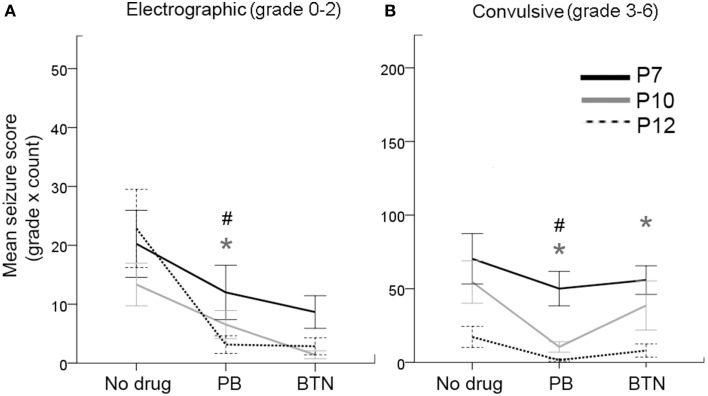
**Behavioral correlates of EEG seizures and PB-efficacy. (A)** Electrographic seizures (grade 0–2) occurred at all ages tested, and responded well to PB treatment at P10 and P12. This response was not significant at P7 (repeated measures ANOVA). **(B)** Likewise, the convulsive seizures (grades 3–6) also showed significant PB-efficacy at P10 and P12 only with significant BTN aggravation of the convulsive seizures at P10 (repeated measures ANOVA). Gray ^*^ = P10, ^#^ = P12, represent significant *p*-values for pairwise *t*-tests. Therefore, PB failed to block either electrographic or convulsive seizures at P7.

The unique advantage of synchronous video-EEG in this study was its ability to identify and quantitate the non-convulsive seizures for the entire data set. Similar to humans, neonatal seizures in rodents can be difficult to identify by behavioral parameters alone (Cuaycong et al., 2011). To evaluate if the treatment efficacy was different for seizures graded as 0–2 (i.e., electrographic—ranging from inactive to behavioral correlates associated with movements not overtly convulsive—see Methods) and 3–6 (i.e., convulsive), the data were grouped by these seizure scores as the sum of [grade of the seizure X count] in each epoch of the recording (Figure 3). Electrographic seizures were detected at every age investigated in this study, and were not significantly different for the probability of occurrence during the baseline EEG (i.e., early seizures in immediate post-stroke period; Figure 3A). Convulsive seizures showed an age-dependent decrease of occurrences for baseline EEG (Figure 3B); however, this difference was not significant. Repeated measures ANOVAs for PB+BTN treatment efficacy showed that the within-subjects drug effects were significantly efficacious for electrographic seizures at P10 and P12 but not at P7 (P7: *df* = 2, *F* = 3.025, *p* = 0.07, P10: *df* = 2, *F* = 5.224, *p* = 0.03, and P12: *df* = 2, *F* = 9.144, *p* = 0.004). However, for grade 3–6 seizures, the within-subjects drug effects for PB+BTN efficacy were not significant at P7 and P12 but significant at P10 (i.e., P7: *df* = 2, *F* = 1.224, *p* = 0.3, P10: *df* = 2, *F* = 6.604, *p* = 0.02, and P12: *df* = 2, *F* = 3.262, *p* = 0.07). For the pairwise *t*-tests, PB significantly dropped the scores of both electrographic and convulsive seizures at P10 and P12, but failed to curb either at P7. In contrast, follow-on BTN treatment did not add any significant therapeutic benefit on electrographic seizure scores at any age and the post-BTN increase of seizure burden at P10 was driven by significant increase in the occurrence of convulsive seizures following effective PB-driven seizure suppression.

### Correlation of baseline seizure severity to post-PB efficacy as a function of post-natal age

**Figure 4:**
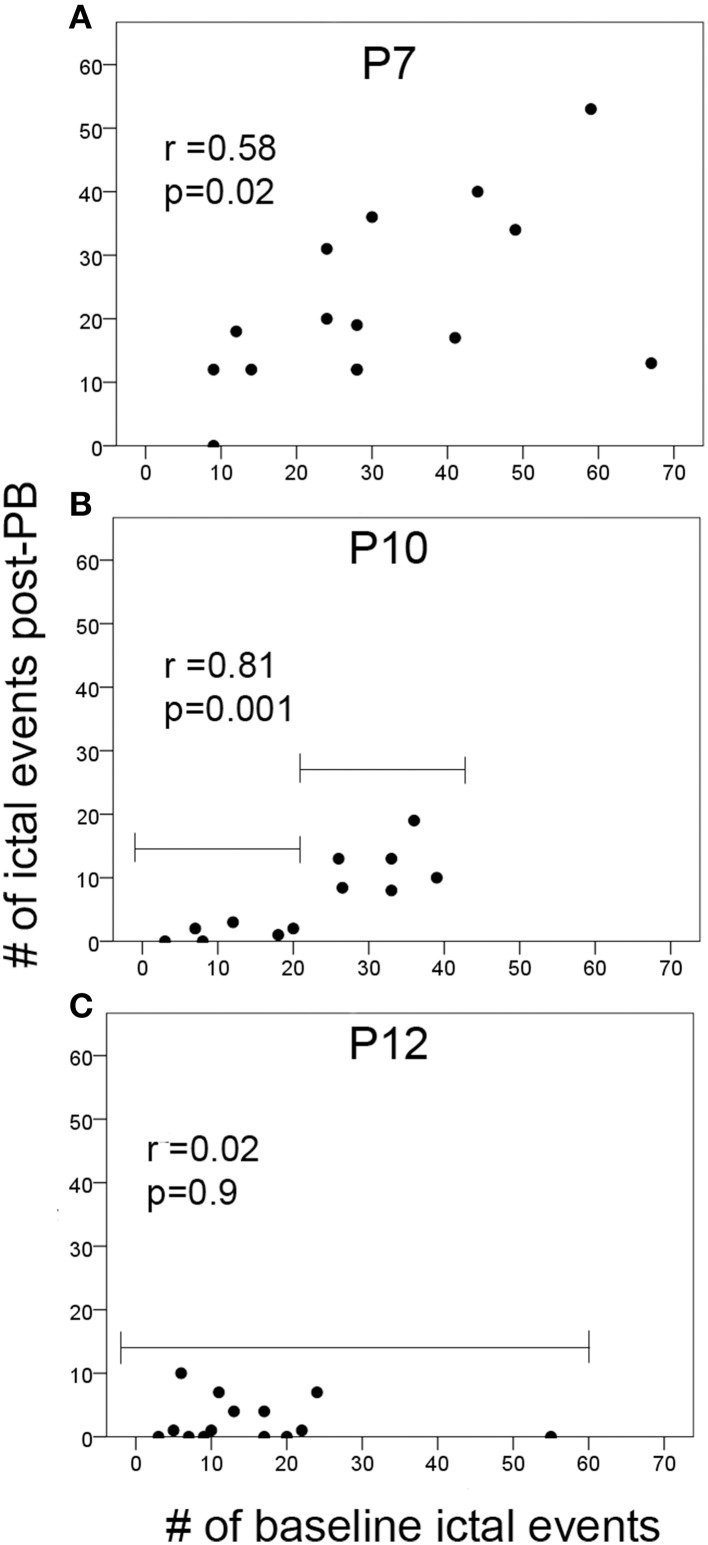
**Ictal events vs. PB-efficacy. (A–C)** Correlations of the number of ictal events before treatment (i.e., during baseline EEG recording) to the number of ictal events post-PB treatment at P7, P10, and P12. Brackets in **(B,C)** show the difference in the PB-efficacy at P10 vs. P12. Post-PB seizure suppression at P12 was consistently significant and uniform regardless of baseline seizure severities (*p* = 0.09). In contrast, at P10, PB-efficacy was significantly dependent on the baseline seizure severity (i.e., better efficacy with lower baseline seizure loads compared to higher baseline seizure loads). Additional correlations run for baseline vs. post-PB seizure burdens showed similar results (see brackets in **B**; first bracket ≤20 i vs. second bracket >20 ictal events); low baseline seizure burdens and PB-efficacy were not significantly correlated (for baseline seizure burden ≤250 s; *r* = 0.35, *p* = 0.39); high baseline seizure burdens showed significant positive correlations to their post-PB seizure burdens (for baseline seizure burden ≤250 s; *r* = 0.61, *p* = 0.03). This may indicate the potential role of the number of ictal events that have occurred before treatment on the efficacy of anti-seizure agents at P10 (*p* = 0.001).

To evaluate whether the severity of seizure burden during baseline EEG with no drug on board contributed to the PB-efficacy given 1 h later, correlations between the numbers of ictal events during baseline recording and during post-PB recording were evaluated in the ligated-treated group (Figure 4). At P7, there was a significant positive correlation between the number of ictal events in the 1st and 2nd post-PB treatment hours (Figure 4A; *p* = 0.02). Since PB was inefficacious at P7, this indicated that PB had no effect on overall post-PB ictal counts in each pup. Interestingly, correlations at P10 after PB-treatment compared to baseline showed a significantly stronger positive correlation than at P7 (Figure 4B; *p* = 0.001). Since PB was an efficacious anti-seizure agent at P10, this may indicate that the anti-seizure efficacy of PB was dependent on baseline seizure burdens (i.e., if initial seizure burden was high, follow-on post-treatment seizure burden remained high). Additionally, Figure 4B, group brackets representing low and high baseline seizure burdens indicate that PB was very efficacious in subduing all low seizure burdens (i.e., ≤250 s). This is supported by additional correlations run for baseline vs. post-PB seizure burdens which showed that low baseline seizure burdens and PB efficacy at P10 were not significant (for baseline seizure burden ≤250 s; *r* = 0.35, *p* = 0.39) but high baseline seizure burdens at P10 showed significant positive correlations to their post-PB seizure burdens (for baseline seizure burden >250 s; *r* = 0.61, *p* = 0.03). In contrast, at P12, baselines vs. post-PB correlations were not significant (Figure 4C). Since PB was highly efficacious at P12, this illustrates a strong anti-seizure efficacy of PB, irrespective of baseline seizure burdens.

### Age-dependent stroke injury

**Figure 5:**
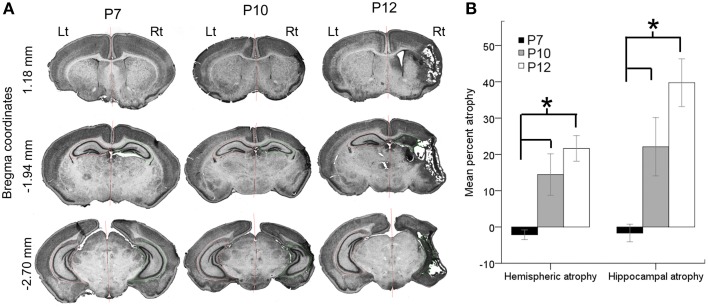
**Age-dependent stroke injury. (A,B)** Severity of the ischemic injury evaluated at P18 in ligated-treated mice. Histopathological analyses were performed on a series of coronal brain sections harvested at P18 for all the ages investigated. Post-ischemic P7 brains were significantly less vulnerable to necrotic infract injury compared to P10 and P12. Both hemispheric and hippocampal atrophies associated with stroke injury were significantly higher at P10 and P12. The stroke severities between P10 and P12 were not significantly different. ^*^p < 0.05.

Stroke injury severities were quantitated at P18 for all brains processed for histology (*n* = 49; P7 = 15, P10 = 15, and P12 = 19) and the remaining brains from study were fresh frozen for western blot analysis. Ligations at P7 did not result in a cystic infarct injury when evaluated at P18 (Figure 5A). Although the P7 ligated brains, when harvested for western blot analysis at 6–8 h after ligation showed edema of the ipsilateral hemisphere, no measurable atrophy was detected when P7 ligated brains were harvested and processed for histology at P18 (Figure 5). Microscopic examination also did not reveal obvious cell death in the watershed zones. Post-stroke diffuse cell death cannot be ruled out in the P7 brains however; compared to the P10 and P12 ligated pups, injury at P7 in CD1 ischemic pups may have white matter injury, which was not evaluated in this study (Fatemi et al., 2011). Overall, P7 pups showed a significant resistance to necrotic infarcts in the middle cerebral artery perfusion territory detected at P10 and P12 (Figures 5A,B; *p* = 0.005 P7 vs. P10 for both hemispheric and hippocampal atrophy and *p* < 0.0001 P7 vs. P12 for both hemispheric and hippocampal atrophy). The mean hemispheric and hippocampal atrophy in P10 were not significantly different from those in P12 (*p* = 0.3 and 0.1, respectively).

To determine whether injury severity evaluated at P18 correlated with the seizure severity in the 1st 3 h after ischemic insult at the three ages examined, hippocampal and hemispheric atrophies were compared to the time spent seizing on EEG. No significant correlations were detected at any age. Additionally, significant positive correlations between hemispheric to hippocampal atrophy were detected for the P12 (*p* = 0.008) ligated group at P18, which has been reported previously for ligated-control P12 mice at P40 (Kadam et al., 2008b). Similar correlations were not detected for the P10 ligated-treated group. No significant correlations were detected between the severities of ischemic seizure burdens at baseline and the severity of the ischemic injury at P18, in either the P10 or P12 pups. No significant sex differences in injury severity were noted at P10 and P12 either. Since stroke injuries evolve over time, injury assessments at longer survival time-points may be more predictive of initial seizure burdens.

### Age- and sex-dependent seizure susceptibilities: is KCC2 a major player?

**Figure 6:**
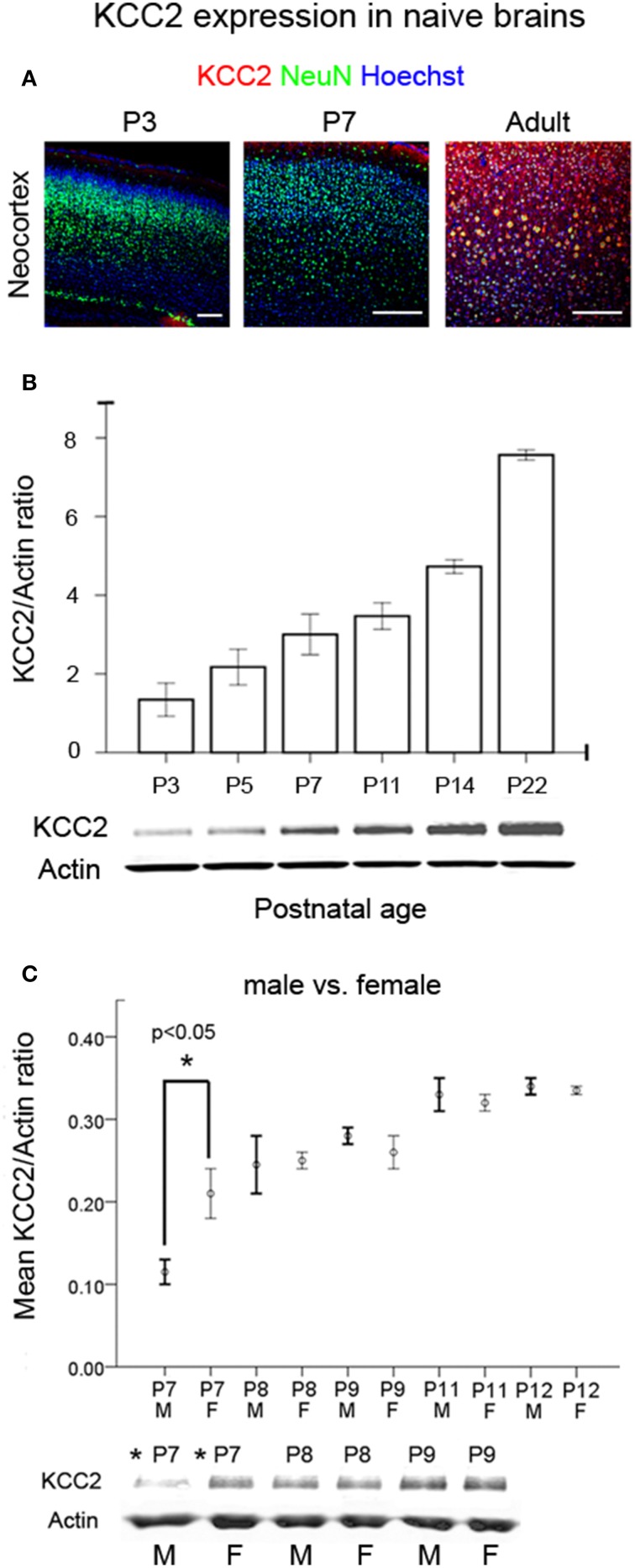
**Developmental profile of KCC2 expression. (A)** IHC for KCC2 in cortex as a function of postnatal age in naïve brains respectively (Scale bar = 250 um) showed increased neuronal expression with age. **(B)** Western blot quantitation of KCC2 expression as a function of postnatal age in naïve brains (*n* = 3 for each age). Bar graphs show the co-transporter expression normalized to actin expression of the same brains. **(C)** A significant sex-dependent lag of KCC2 expression was detected at P7 in naïve males compared to age-matched naïve females (^*^p < 0.05), and this dimorphism was not significant at older ages [M, male; F, female (*n* = 2 each at every age)]. Analyses for NKCC1 in the same brain samples are shown in Supplementary Figures 4A–C.

**Figure 7:**
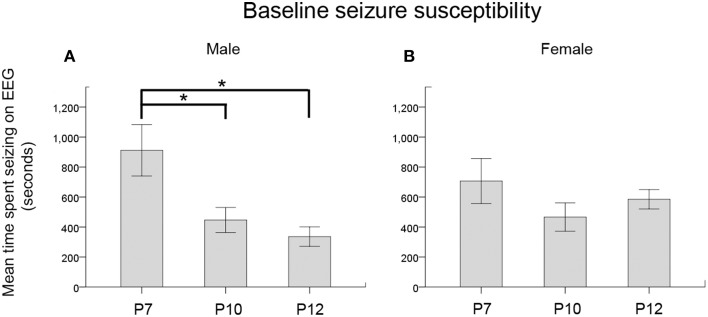
**Seizure severity by age and sex. (A,B)** Baseline seizure burdens pooled for ligated-control and ligated-treated pups showed an age-dependent susceptibility for ischemic seizures that was significant in males but not in females. ^*^p < 0.05

To establish an age-dependent expression profile for the CD1 mouse strain used in this model, we examined KCC2 and NKCC1 expression in naïve pup brains in ages advancing from P3 to P22 (Figure 6A and Supplementary Figure 4A). As reported previously in both rodents and humans (Dzhala et al., 2005), we saw an age-dependent increase in the expression of KCC2 examined both by IHC and western blot analyses (Figures 6A,B). In contrast, NKCC1 [i.e., detecting NKCC1a, the non-dominant spice-isoform (see Methods Section—Histology)] showed an age-dependent decrease using the same samples (Supplementary Figures 4A,B). Additionally at P7, a sex-dependent lag in the KCC2 expression levels was detected in naïve males compared to the age-matched females, which was not detected at older ages when examined up to the age of P12 (Figure 6C). Similar sex-dependent maturational lags have been previously reported for males (Nunez and McCarthy, 2007; Perrot-Sinal et al., 2007; Galanopoulou, 2008; McCarthy et al., 2012; Murguia-Castillo et al., 2013). No sex-dependent maturational lags were detected with NKCC1 (Supplementary Figure 4C). These findings, in addition to the higher susceptibility to ischemic seizures detected in P7 males (see Figures 7A,B) may indicate KCC2 as the major player underlying the age-dependent seizure susceptibility detected in this model.

### Sex-dependent susceptibility to ischemic seizures and response to anticonvulsants

When the baseline EEG scores (i.e., seizure burden in the 1st h post-ligation) for both ligated-control and ligated-treated were pooled to further analyze the effect of sex on the age-dependence of seizure susceptibility, a One-Way ANOVA showed a significance for males only. *Post-hoc* (Bonferroni) comparisons showed P7 males to be significantly more susceptible to seizures than either P10 (*p* = 0.04) or P12 (*p* = 0.01) males (Figure 7A, Supplementary Figures 2B,C). No significant differences were noted for females (Figure 7B). Based on this finding, future studies need to address this sex and age-dependent ischemic-seizure susceptibility when evaluating the efficacy of PB and BTN treatment.

### PB-resistant ischemic seizures and an acute downregulation of KCC2 expression

**Figure 8:**
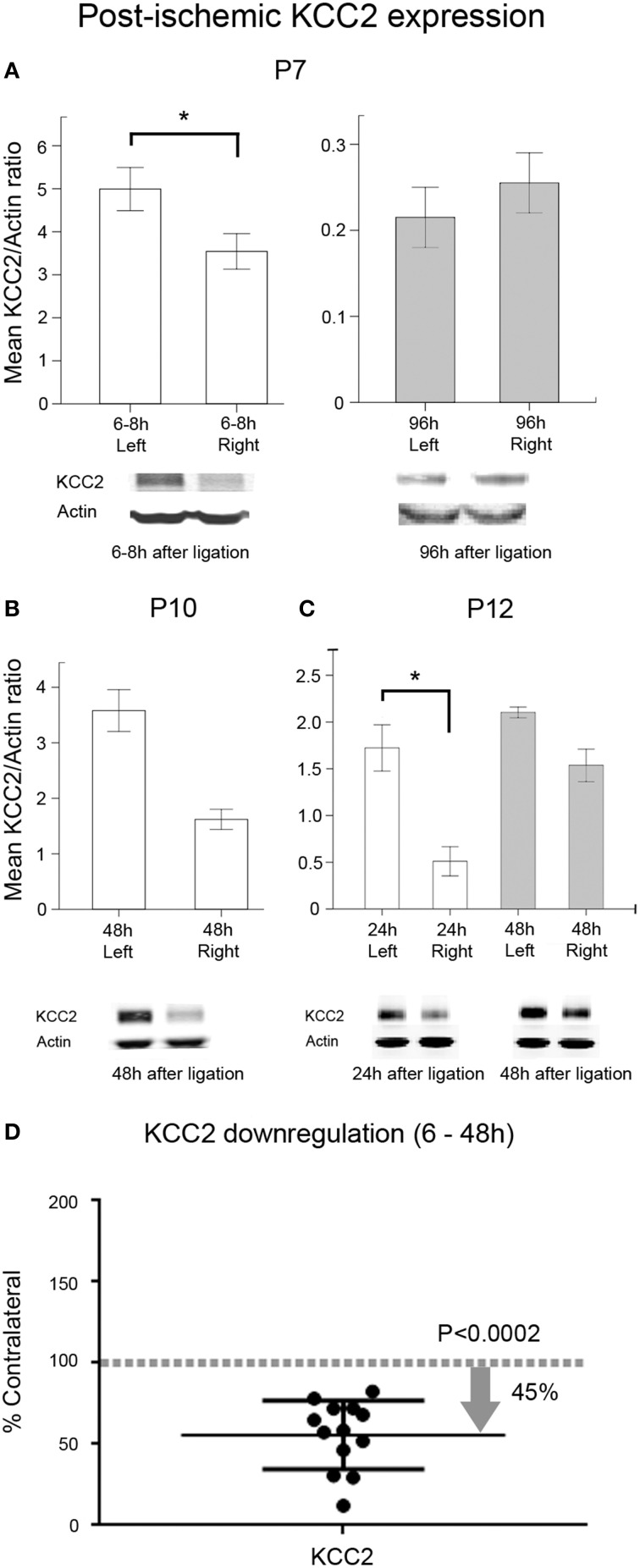
**Post-ischemic KCC2 downregulation**. Western blot quantification of post-ligation expression of KCC2 in P7, P10, and P12 ligated pups at acute and sub-acute time-points after ischemia (*n* = 3 each) in ipsi- and contralateral (i.e., injured and uninjured hemispheres, respectively) hemispheres; **(A)** At P7, the significant downregulation of KCC2 detected within 6–8 h of ligation (^*^p = 0.004) showed a complete recovery by 96 h. **(B)** At P10, KCC2 downregulation showed the same trend as at P7 at 48 h. **(C)** P12 pups showed a significant downregulation of KCC2 at 24 h (^*^p < 0.02). **(D)** Scatter plot of KCC2 expression levels, normalized to actin and shown as the percent of levels in their respective contralateral uninjured hemispheres. All acute time-points (6–48 h) were pooled from all three age groups (*n* = 13). Data show that ischemia in the CD1 mice results in an acute KCC2 downregulation in the ischemic injured hemispheres with approximately 45.35% reduction in mean expression (pairwise *t*-test, *p* = 0.0002; contralateral control is 100%, which is represented as a dotted gray line as a ratio of 1).

In this study, an acute post-ischemic downregulation of KCC2 expression was detected in the ipsilateral hemisphere compared to the contralateral hemisphere beginning from a few hours to 48 h after ischemia at all ages tested (Figures 8A–D). The post-ischemic downregulation of KCC2 expression in ipsilateral hemisphere was ~45% compared to uninjured contralateral hemisphere (Figure 8D; pairwise *t*-test, *p* = 0.0002). A trend toward recovery from the downregulation was also detected in the P7 age group at 96 h after ischemia (Figures 8A–D). Although in humans NKCC1a represents a non-dominant isoform of NKCC1 splice variant, the analogous expression profiles in rodents are not known due to the current lack of a pan-NKCC1 antibody. Using the currently available antibodies, our data matched the previously published data where NKCC1 was shown to decrease with advancing age in naïve brains (Supplementary Figure 4B). There were paradoxical trends toward post-ischemic increase in NKCC1 levels in the ipsilateral hemisphere at ≤48 h in this study (Supplementary Figure 5D), as it was shown in a lesion study (Shimizu-Okabe, 2007). Similar findings have also been noted after neonatal hypoxia-ischemia (Dai et al., 2005). These results indicate that neonatal ischemia significantly alters the acute and sub-acute developmental profiles of the adult-form chloride transporter KCC2; however, NKCC1 developmental expression profiles remain relatively unaltered or increased. While ischemia-related changes in cellular populations were expected with the stroke lesions, both co-transporters, KCC2 and NKCC1 would be similarly affected by these changes at the three age groups evaluated (i.e., P7 group where no infarct lesions were seen at P18 vs. P10 and P12 where they were commonly detected; see Figure 5). Both of these co-transporters were evaluated in the same homogenized brain samples, and KCC2 showed consistent downregulation at 6 to 48 h and recovery at 96 h. More importantly, the finding of PB-resistance at P7 and the seizure-burden dependent efficacy at P10 validates the P7-P10 CD1 mouse model of neonatal ischemia reported here as a novel tool to test the efficacy of novel anti-seizure pharmacotherapies in a clinically relevant model of seizure induction.

## Discussion

This study has several salient findings to report. (1) A new mouse model of ischemic seizures that developed both primarily PB-resistant seizures at P7 and PB-responsive seizures at P10. At P10, PB-efficacy was dependent on the baseline seizure burden (i.e., lower the seizure burden better the anti-seizure efficacy). The acute seizures recorded in the first 3 h after initiation of ischemia represented a status-like seizure burden state well described for severities typically seen clinically in HIE (Boylan and Pressler, 2013). (2) The age-dependent seizure susceptibility after ischemia was significantly higher at P7 than both at P10 and P12. This susceptibility may represent the underlying age-dependent upregulation of KCC2 expression in maturing brains. (3) An acute and significant post-ischemic KCC2 downregulation was detected at all ages tested. The post-ischemic KCC2 downregulation catches up with the age-dependent developmental increase, representing recovery from ischemic insult within a few days. Therefore, ischemic injury significantly modulates the developmental profile of the adult-form chloride co-transporter KCC2, and thus dictates the efficacy of anti-seizure medications that follow. (4) The NKCC1 antagonist, BTN, failed to act as an adjunct in the new model for the primary PB-resistant seizures. Additionally, BTN blunted the anti-seizure efficacy of PB treatment at P10 with the follow-on treatment paradigm, by aggravating the PB-subdued seizures. Hence, NKCC1 blockage fails to rescue ischemic seizures regardless of the anti-seizure efficacy of GABA_A_ agonists which fail at P7 but work at P10 and P12. (5) The sex-dependent seizure susceptibility detected in P7 males may correlate with the developmental lag of KCC2 expression in naïve males compared to females at that age. This lag goes away with advancing age.

Neonatal seizures, especially those associated with ischemia, are known to be transient in the neonatal period. HIE seizures also show hours of increasing seizure burdens alternating with quiet or low-seizing periods with crests and troughs during their natural temporal progression (Low et al., 2014). How aggressively we treat these transient seizures, which may be severe in some cases, with drugs that may also alter the developmental profile of an immature brain, is a subject of debate (Kossoff, 2011). The exact time of onset of ischemia in neonates is rarely known, and clinical seizures are detected a few hours to days later. This may be due to either, a failure to detect the subtle early seizures since most of the EEG seizures are non-clinical (Low et al., 2014), or a slower paced evolution of the ischemic injury. The dynamics of this evolution is however poorly understood and difficult to quantitate clinically. Recent studies (Kwon et al., 2011) that have tried to evaluate the issue are difficult to interpret with regards to evolution because the baseline seizure burdens before onset of treatment (Nash et al., 2011; Wusthoff et al., 2011) are rarely known or quantifiable without EEG (Low et al., 2014). This limitation in reported clinical studies persists due to the nature of the disease and the lack of EEG data to accurately assess pre-treatment seizure burdens of non-clinical seizures. Even so, PB-inefficacy as first-line treatment is now widely reported (Painter, 1999; Sankar and Painter, 2005).

Intrinsic features of immature networks make GABA_A_-based pharmacotherapy more difficult (Rakhade and Jensen, 2009). Following recurrent seizures, it has been shown that intracellular chloride ions accumulate, making GABA strongly excitatory (Khalilov et al., 1999; Khazipov et al., 2004; Khirug et al., 2005; Nardou et al., 2011). Recent research has shown that KCC2 downregulation following excitotoxic injury may underlie these findings (Nabekura, 2002; Stein, 2004; Wake et al., 2007; Gomes et al., 2012). Developmentally, NKCC1 mediates influx of chloride ions (Dzhala et al., 2010); however, this chloride co-transporter is neither necessary nor sufficient, as these shifts of GABA polarity also occur in NKCC1 KOs (Nardou et al., 2011; Ben-Ari, 2012). Additionally, recent study has identified two spice variants of NKCC1 in the human brain, NKCC1a [1-27] and NKCC1b [1-27 (Δ21)] (Morita et al., 2014). NKCC1b is the dominant splice variant in human brains, which shows an age-dependent upregulation (Kang et al., 2011): Human Brain Transcriptome website. No reliable pan-NKCC1 antibodies, capable of detecting both variants, are currently available for reevaluating the NKCC1 data reported here (Supplementary Figures 4, 5) or the similar published data in animal models that may have only quantitated NKCC1a, which was shown to downregulate with advancing age (Plotkin et al., 1997; Dzhala et al., 2005; Aronica, 2007). The activity-dependent downregulation of KCC2 after NMDA-induced excitotoxicity may lead to appearance of PB-resistance (Lee et al., 2011; Puskarjov et al., 2014a), and pre-clinical animal models of neonatal seizures that do not result in KCC2 downregulation may not be relevant to ischemic seizures. Reports of KCC2 downregulation in the white-matter of premature babies with white matter lesions support this hypothesis (Robinson et al., 2010). Even in adult models of epilepsy (Deisz et al., 2011, 2014; Campbell et al., 2015), downregulation of KCC2 has been detected in human cortices resected for refractory seizures and in peritumoral neurons in mouse cortical slices, further confirming that KCC2 is the key player in maintaining chloride homeostasis in mature neurons. The expression profile of the co-transporters in HIE brains remains unknown and has the potential to add significant insights into pre-clinical animal modeling (Kang and Kadam, 2014). Therefore, preventing KCC2 downregulation following injury may help delay the intracellular chloride accumulation during repetitive ischemic seizures and thus increase the efficacy of GABA_A_-agonists. The critical role of KCC2 for chloride homeostasis and ultimately neuronal survival has been well established in KCC2 knockout (KO) model of *in vitro* and *in vivo* (Hubner, 2001; Pellegrino et al., 2011). Knockout of isoforms, KCC2a and b are lethal in mice due to respiratory failure, while KCC2b KO mice can survive up to P17, with frequent and severe spontaneous seizures (Woo, 2002; Blaesse et al., 2009). In addition, the now known role of KCC2 in spine development (Li et al., 2007) and cortical interneuron migration (Bortone and Polleux, 2009) may indicate that post-excitotoxic downregulation of KCC2 may underlie the development of long-term sequelae like cognitive and behavioral deficits. In contrast, the NKCC1 KO mouse does not have spontaneous seizures, is non-lethal but deaf (Flagella et al., 1999). Since our study showed a strong correlation between the severity of the early untreated ischemic seizures and PB-efficacy as a function of age, this animal model of PB-resistant seizures would be a useful tool to test this hypothesis further.

Using physiologic techniques, the intracellular chloride shift has been shown to be primarily due to the downregulation and internalization of the chloride exporter KCC2 (Lee et al., 2010, 2011; Nardou et al., 2011). It has been shown that KCC2 downregulation occurs immediately within minutes after an excitotoxic injury (Rivera et al., 2004; Puskarjov et al., 2012). This finding complements data from other studies showing that this co-transporter is highly sensitive to serine and tyrosine phosphorylation and seizures that control its turnover (Lee et al., 2010, 2011; Kahle et al., 2013). The diuretic NKCC1 antagonist, BTN, has been proposed as a novel anti-seizure medication (Dzhala et al., 2008) and was the basis of a current clinical trial (NEMO, FP7-EU clinical trial: http://www.nemo-europe.com/ and ClinicalTrials.gov; NCT00830531); however, BTN blocks seizures in some but not all models of seizures (Kilb et al., 2007). Even with a pre-treatment protocol used in a chemoconvulsant model in neonatal rats, studies have shown an age-dependent specificity of the lack of efficacy of BTN (Mares, 2009) where higher doses actually decreased the latency to generalized seizures in the older pups (i.e., P12). With the recent reports of the termination of the European clinical trial reporting BTN-inefficacy for HIE seizures associated with ototoxicity (Pressler et al., 2015), testing higher doses of BTN seems counterproductive. The results of our experiments using a clinically relevant post-treatment protocol support these reports. In the immature brain, it is possible that the very early seizures, which occur before a significant KCC2 downregulation has begun, may be efficaciously blocked by BTN alone or as an adjunct treatment after PB (Nardou et al., 2009, 2011). In addition, BTN as an adjunct treatment to PB may work efficaciously in neonatal seizures that are not associated with KCC2 downregulation (Cleary et al., 2013). However, after recurrent seizures and KCC2 degradation (Puskarjov et al., 2015), GABA strongly excites neurons in the immature brain, and drugs like PB that act as GABA_A_ agonists fail to act. Similarly, our findings suggest that PB-efficacy is dependent on the baseline seizure burdens specifically at P10 in our model. Our studies show that BTN fails to improve PB-efficacy when given as a follow-on treatment at P7. Our results also show that BTN can blunt PB-subdued seizures at P10. The BTN aggravation in our study following PB-efficacy may not be detected clinically, since BTN would not be administered to a patient whose seizures have responded well to PB. However, this finding is of scientific significance and deserves further evaluation to understand the underlying mechanism.

In general, the use of BTN in critically ill patients requires caution. Blocking NKCC1 function in the brain during development may interfere with critical circuit formation (Wang and Kriegstein, 2010). BTN non-specificity as a NKCC1 antagonist and its ability to also block KCC2 at higher doses (Puskarjov et al., 2014b) should be an additional concern in a seizing brain. The associated ototoxicity detected in the NEMO trial associated with the expression of the same NKCC1 isoform in the inner hair cells, should raise caution for all neonatologists who use BTN for its approved use as a diuretic (Pressler et al., 2015). In a model of hypoxic seizures, the maximum concentration of BTN in the brain was estimated to be 1.2 ng/ml following an acute dose of BTN injected IP at 0.3 mg/kg (Cleary et al., 2013). Recent review also discusses the various reasons for the low bioavailability of BTN in brain, a significant caveat to this line of pharmacotherapy (Puskarjov et al., 2014b). Although BTN prodrugs designed for better brain bioavailability now exist, the anti-seizure efficacies of those BTN prodrugs were recently investigated and show no clear effect (Tollner et al., 2015). Additionally, the issue of side-effects such as diuresis and ototoxicity remain. The phenomenon of BTN aggravation of seizures reported here occurred only after PB was efficacious in subduing the ischemic seizures (Supplementary Figures 3,6) in an immature brain where KCC2 is already significantly downregulated. In addition to the acute side-effects, BTN has also been shown to result in deleterious long-term effects. A significant increase in the percentage of rats having spontaneous seizures has been reported in response to the acute administration of BTN following PB in a pilocarpine model of temporal epilepsy (Brandt et al., 2010). BTN may also attenuate the seizure-induced activation of HPA by blocking NKCC1 in periventricular neurons (O'Toole et al., 2014). However, this study was done in adult rodents, and the effect of BTN on HPA axis in immature brains is not known. Overall, further work is required to understand the potential cause of post-BTN aggravation of seizures reported in this model.

Early and effective treatment of neonatal seizures following excitotoxic insults is of course the gold standard for management; but early and efficacious treatment is not always attainable. Inability to detect the exact onset of ischemia, failure to detect subtle neonatal seizures, and the likelihood of preceding *in utero* ischemic insults may lead to the early KCC2 downregulation and a lag in developmental profile that may underlie the increased seizure susceptibility and PB-resistance of the first detected seizures. Ischemia in the developing brain likely alters the expression profile of a multitude of factors that modulate transmembrane ionic gradients. This study shows that the pathology underlying the occurrence of neonatal seizures may dictate drug responses. Current clinical trials for BTN were initiated based on earlier reports of BTN-efficacy from non-ischemic models that may depend on the stable KCC2 expression following the initial insult. However, our data show that KCC2 downregulation, beginning *in utero* following ischemia or possibly infection/inflammation, may make early interventions with BTN futile. In conditions where KCC2 is already downregulated before birth or lagging in the age-dependent upregulation (Morita et al., 2014), blocking Cl^−^ import through NKCC1 cannot compensate for the role of KCC2 as a Cl^−^ extruder. Additionally, we do not clearly understand the BTN-induced aggravation of seizures detected in our study that was also differentially modulated by sex. BTN half-life in rodents is short and at ~ 30 min (Cleary et al., 2013), clearly less than the 1 h of seizure burden quantitated in this study. BTN aggravation, when noted, began within 5–10 min of the treatment and lasted throughout the 1 h of recording (Supplementary Figures 3, 6). Recent evidence of estradiol modulation of NKCC1 (Nugent et al., 2012) shows that many details of this process and its sex-dependent modulation remain to be understood. The overall age-dependent susceptibility of males to ischemic seizures in this study and the additional window period of a lag in KCC2 development in naïve brains may add to the accumulating data on male susceptibility to developmental injury and differential effects of neonatal treatments by sex (Johnston and Hagberg, 2007).

In conclusion, this study highlights the variability of drug responses in animal models based on the mechanism by which the seizures are induced. For BTN, these model-specific outcomes have already been reported to be very variable (Kilb et al., 2007). Dose-dependent efficacy detected for higher doses of BTN (Mazarati et al., 2009) has been shown to suppress neonatal kindling when given as a pre-treatment protocol and has been proposed as a reason behind the failure of lower dose regimens reported in other models. However, pre-treatment protocols have also resulted in an aggravation of seizure onset latencies with higher doses in immature rats using chemoconvulsants (Mares, 2009). Additionally, BTN failed to work at P7 in that study which highlights the age-dependency for its efficacy. The findings of our study suggest that when KCC2 levels remain unaltered or become enhanced after kindling, such protocols may not be translationally relevant to the HIE patients being recruited in the current clinical trials where ischemia is the prominent underlying cause. This hypothesis is now supported by outcomes reported in the recently published NEMO study (Pressler et al., 2015). The same caveat would apply to the translational value for the BTN pre-treatment paradigms that alter the chloride gradients in the naïve brains prior to insult induction in a dose-dependent manner. The variable effects of BTN reported in recent literature and in this current study, highlight the need for further translational research using BTN (Vanhatalo et al., 2009). A recent study, Cleary et al. has reported beneficial dose-dependent effects of PB+BTN pre-treatment in a rat model of hypoxic neonatal seizures (Cleary et al., 2013). Since they also reported a significant upregulation of KCC2 in hippocampus on the day of assessment of BTN treatment efficacy, these data strongly suggest that KCC2 downregulation after ischemia may be a major player in the development of PB-resistance. BTN efficacies were shown to be age- and sex-specific even within the relatively narrow age range investigated in this study. None of the pre-treatment studies noted above examined sex differences. Our results also indicate that neonatal stroke/seizures left undetected or untreated for extended periods of time alter the acute and sub-acute developmental profiles of the adult-form chloride co-transporter KCC2 such that the later seizures may become resistant to treatment with the conventional anticonvulsants that act as GABA_A_ agonists (Puskarjov et al., 2014b, 2015). Prolonged seizures are also known to alter GABA_R_ such that it reduces the efficacy of AEDs (Sankar et al., 2002; Jones-Davis and Macdonald, 2003). In neonates, the transient downregulation and the developmental lag in the KCC2 expression profile may additionally result in chronic alteration of the way the immature brain is getting wired within that critical developmental window (Lombroso, 2007). This study shows that a novel focus on preventing KCC2 downregulation or enhancing KCC2 function following neonatal insults may be critical in guiding future approaches for treating PB-resistant seizures in neonates.

### Conflict of interest statement

The authors declare that the research was conducted in the absence of any commercial or financial relationships that could be construed as a potential conflict of interest.

## Footnotes

^1^BTN clinical trial in Europe (2009) http://cordis.europa.eu/projects/241479.

^2^BTN clinical trial in USA (2009) http://clinicaltrials.gov/ct2/show/NCT00830531?term=bumetanide&rank=1.

## Further information

HUMAN BRAIN TRANSCRIPTOME: hbatlas.org/

## Supplementary material

Supplementary Figure 1 **Representative electrographic seizure traces**. EEG traces (panels 1, 2) of visually identified electrographic seizures (associated video behavioral grades 2 and 4). Raw EEG traces are followed by high and low frequency band pass filtered data. Seizure activity in 13–60 Hz range did not occur during non-ictal events (panel 3) associated with movement artifacts or jittery walking activity in non-seizing pups. These data highlight the importance of EEG as the gold standard for seizure burden assessment in the mouse model of neonatal seizures. The behavioral correlates alone were not a reliable measure in evaluating the total seizure burden in pups.

Supplementary Figure 2 **Representative time-compressed plot (1 h) of EEG traces recorded from P7 pups and an additional expanded time scale of a 5 min EEG trace from the same recording. (A)** P7 sham-operated control that was exposed to the same amount of isoflurane anesthesia as other ligated pups; **(B,C)** P7 Representative male and female pup showing higher baseline seizure susceptibility for males following ischemia.

Supplementary Figure 3 **Representative time-compressed plot (1 h) of EEG traces recorded from P7 vs. P10 ligated pups (time-course schematic of drug administration shown at the top)**. An additional expanded time scale of a 5 min EEG trace from the same recordings show individual ictal events. Both EEG traces for P7 and P10 were selected from the median of the each cohort.

Supplementary Figure 4 **Developmental profile of NKCC1 expression. (A,B)** Western blot quantification of post-ligation expression of NKCC1 in P7, P10, and P12 ligated pups at acute and sub-acute time-points after ischemia (*n* = 3 each) in ipsi- and contralateral (i.e., injured and uninjured hemispheres, respectively) hemispheres; **(C)** No lag in the NKCC1 expression was detected in the male vs. female pups at P7 or at later ages in the naïve brains [M, male; F, female (*n* = 2 each at every age)].

Supplementary Figure 5 **Post-ischemic NKCC1 expression**. Western blot quantification of post-ligation expression of NKCC1 in P7, P10, and P12 seizing pups at different acute and sub-acute time-points after ligation in bilateral hemispheres (*n* = 3 each) **(A)** At P7, NKCC1 remained unaltered at 6–8 h with significant increase in expression at 96 h (*p* = 0.049). **(B)** At P10, NKCC1 expression at 48 h post-ligation did not change significantly (*p* = 0.06). **(C)** NKCC1 remained unaltered in the same brains of P12. **(D)** Scatterplot of NKCC1 expression normalized to contralateral uninjured hemisphere. *n* = 13 in Figure 8D and Supplementary Figure 5D (*n* pooled for time-points 6–48 h post-ischemia). Please refer to Figure 8D for further details.

Supplementary Figure 6 **Representative frequency histogram of ictal events after PB+BTN treatment at P7, P10, and P12**. Frequency histograms of EEG identified ictal events depicted as 1 h slots for the 3 h recording period (top to bottom panel) for both P7 and P10 ligated-control and ligated-treated pups. The pups represent mean seizing rates for the data set depicted in Figure 2. The ictal event distribution graphs represent the lack of seizure clustering during the 3 h recording period for the model. The post-BTN 3rd h recording in a P10 pup also represents the age-specific BTN-induced blunt of PB-subdued seizures in the 3rd h of recording. BTN pharmacokinetics are known to be short (≤30 min; Cleary et al., 2013), but the BTN aggravation persisted for the entire 1 h duration of EEG seizure burden.

Supplementary Video 1 **Close up view of convulsive grade 3 behavior with clockwise circling toward the side of ischemia and tonic posturing of right forearm**. This behavior was never recorded in non-seizing or naïve age-matched pups.

Supplementary Video 2 **Close up view of convulsive grade 5 behavior in a ligated pup**.

## Abbreviations

PB: Phenobarbital
BTN: Bumetanide
GABA: gamma-aminobutyric acid
EEG: electroencephalogram
HIE: hypoxic ischemic encephalopathy
AED: anti-epileptic drug.
