# Supplementary figures and images for: Age- and sex-dependent susceptibility to phenobarbital-resistant neonatal seizures: role of chloride co-transporters
Source: Front Cell Neurosci. 2015 May 12;9:173. doi: 10.3389/fncel.2015.00173 (PMC4429249; doi:10.3389/fncel.2015.00173)

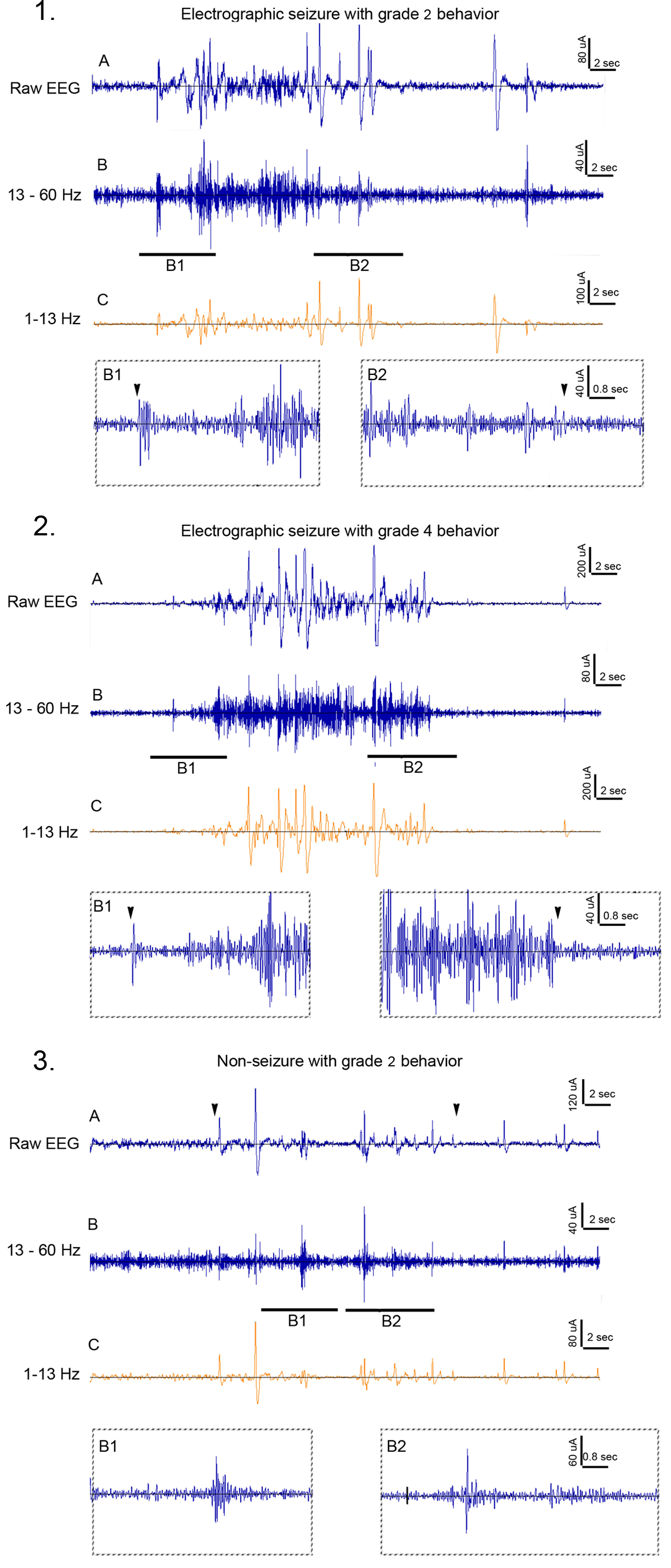

Supplement: Supplementary Figure 1 — Representative electrographic seizure traces. EEG traces (panels 1, 2) of visually identified electrographic seizures (associated video behavioral grades 2 and 4). Raw EEG traces are followed by high and low frequency band pass filtered data. Seizure activity in 13–60 Hz range did not occur during non-ictal events (panel 3) associated with movement artifacts or jittery walking activity in non-seizing pups. These data highlight the importance of EEG as the gold standard for seizure burden assessment in the mouse model of neonatal seizures. The behavioral correlates alone were not a reliable measure in evaluating the total seizure burden in pups. [file Image1.TIF]

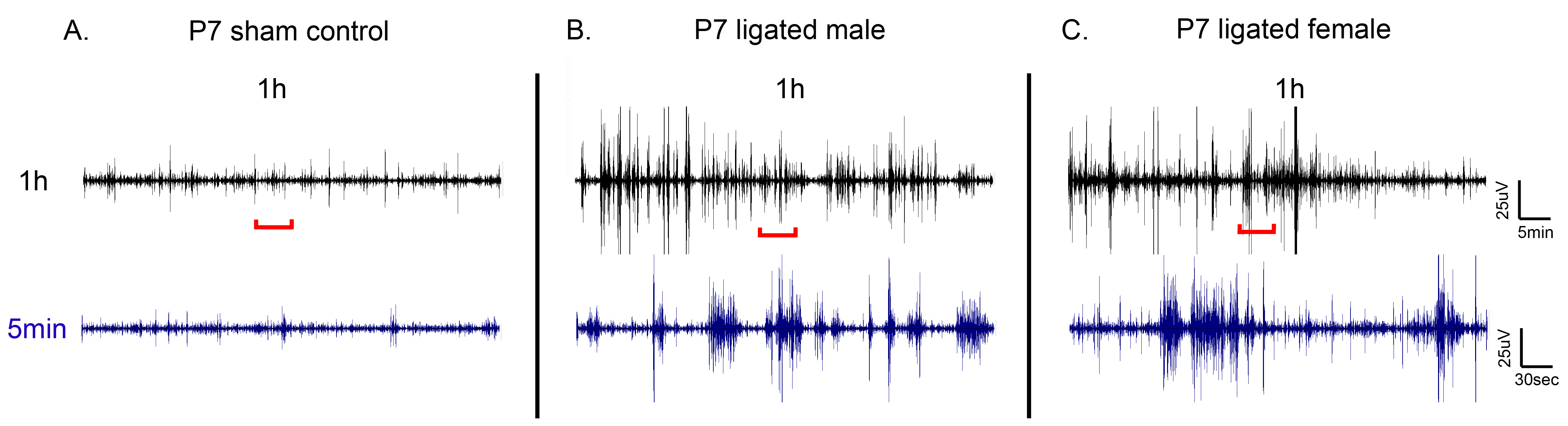

Supplement: Supplementary Figure 2 — Representative time-compressed plot (1 h) of EEG traces recorded from P7 pups and an additional expanded time scale of a 5 min EEG trace from the same recording. (A) P7 sham-operated control that was exposed to the same amount of isoflurane anesthesia as other ligated pups; (B,C) P7 Representative male and female pup showing higher baseline seizure susceptibility for males following ischemia. [file Image2.TIF]

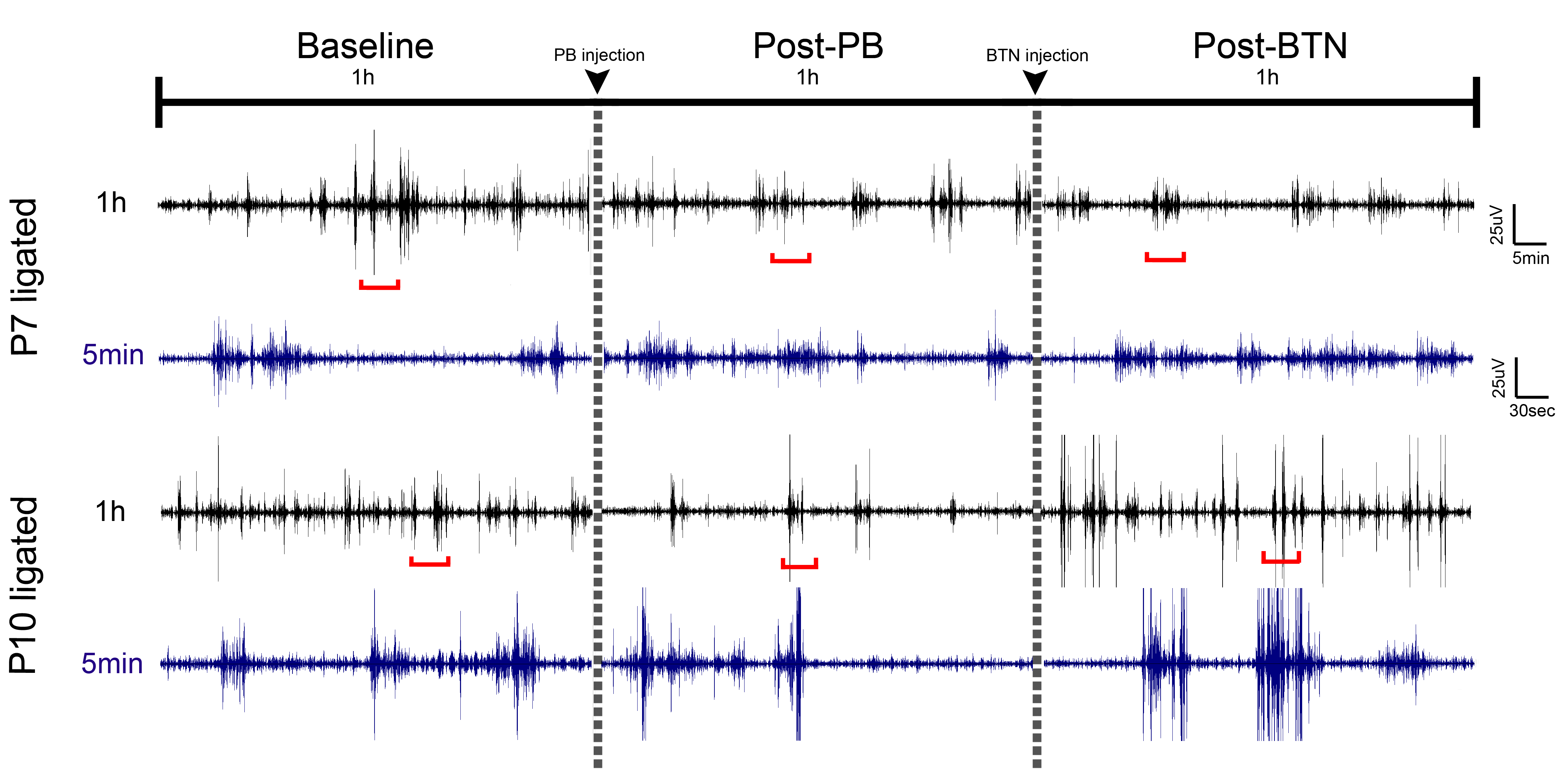

Supplement: Supplementary Figure 3 — Representative time-compressed plot (1 h) of EEG traces recorded from P7 vs. P10 ligated pups (time-course schematic of drug administration shown at the top). An additional expanded time scale of a 5 min EEG trace from the same recordings show individual ictal events. Both EEG traces for P7 and P10 were selected from the median of the each cohort. [file Image3.TIF]

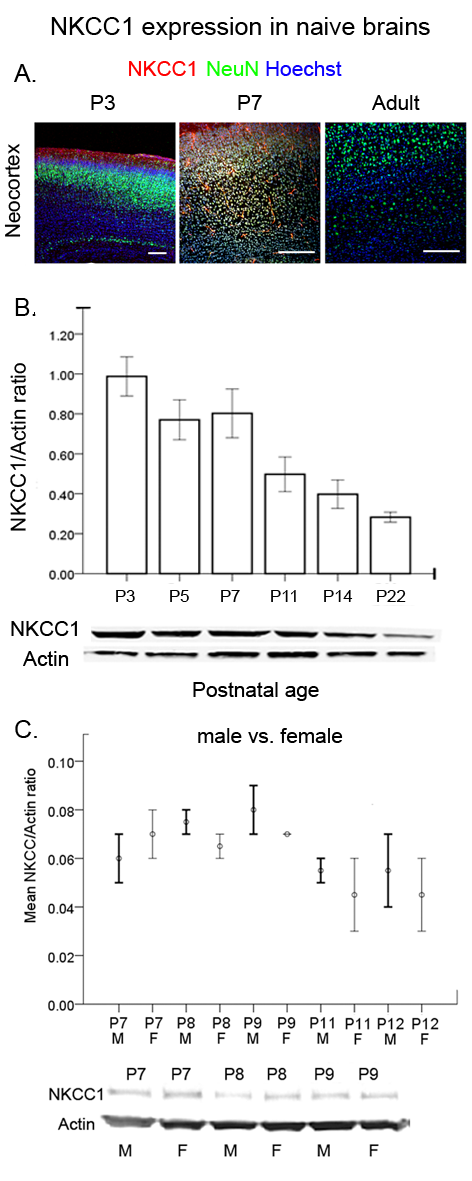

Supplement: Supplementary Figure 4 — Developmental profile of NKCC1 expression. (A,B) Western blot quantification of post-ligation expression of NKCC1 in P7, P10, and P12 ligated pups at acute and sub-acute time-points after ischemia (n = 3 each) in ipsi- and contralateral (i.e., injured and uninjured hemispheres, respectively) hemispheres; (C) No lag in the NKCC1 expression was detected in the male vs. female pups at P7 or at later ages in the naïve brains [M, male; F, female (n = 2 each at every age)]. [file Image4.TIF]

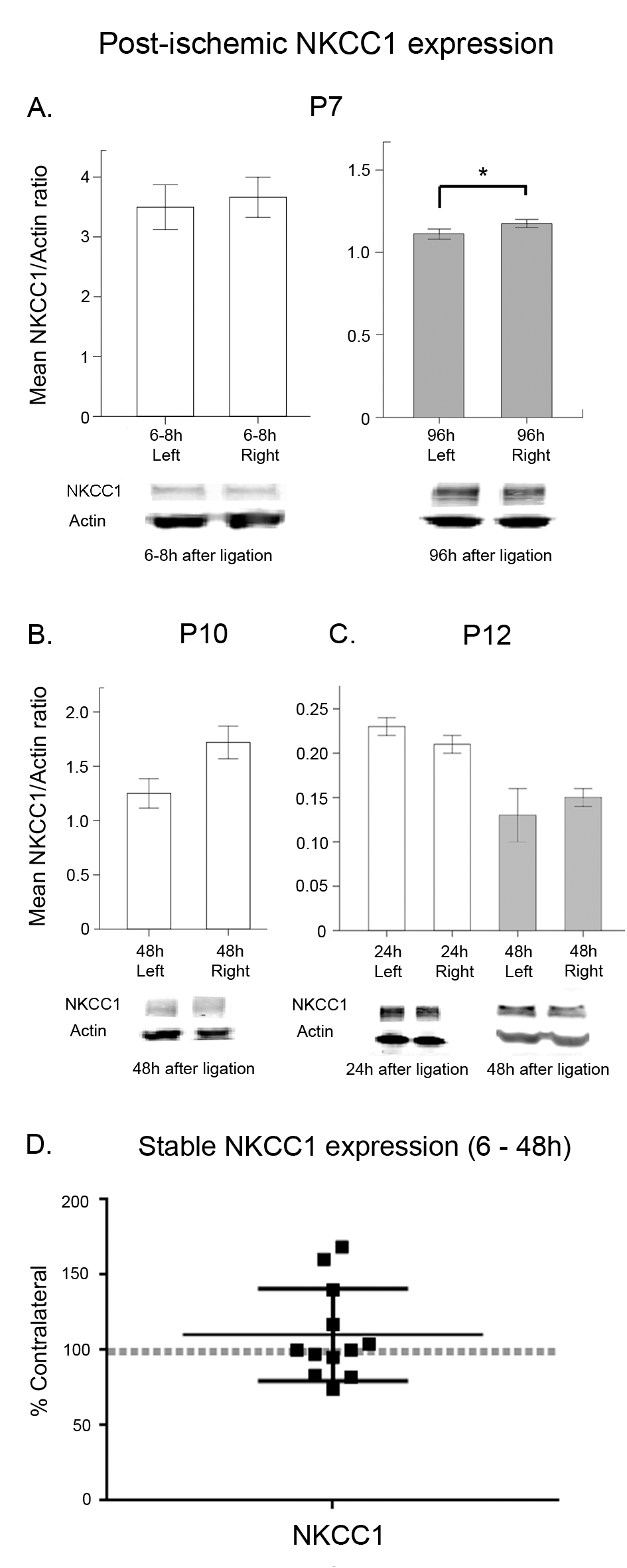

Supplement: Supplementary Figure 5 — Post-ischemic NKCC1 expression. Western blot quantification of post-ligation expression of NKCC1 in P7, P10, and P12 seizing pups at different acute and sub-acute time-points after ligation in bilateral hemispheres (n = 3 each) (A) At P7, NKCC1 remained unaltered at 6–8 h with significant increase in expression at 96 h (p = 0.049). (B) At P10, NKCC1 expression at 48 h post-ligation did not change significantly (p = 0.06). (C) NKCC1 remained unaltered in the same brains of P12. (D) Scatterplot of NKCC1 expression normalized to contralateral uninjured hemisphere. n = 13 in Figure 8D and Supplementary Figure 5D (n pooled for time-points 6–48 h post-ischemia). Please refer to Figure 8D for further details. [file Image5.TIF]

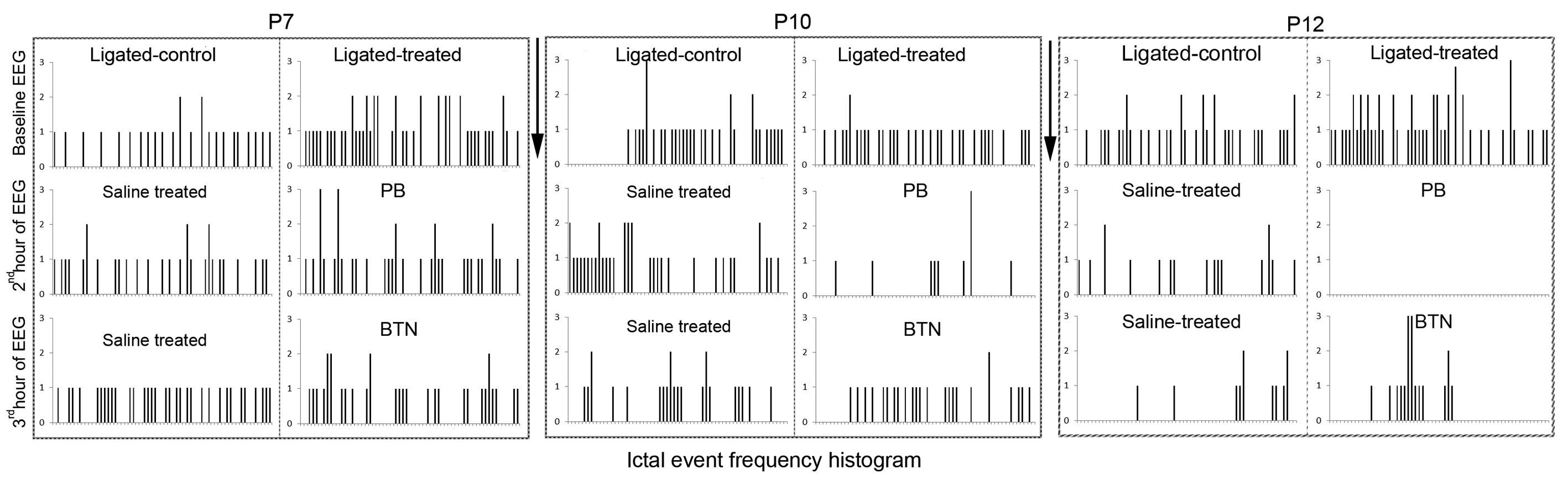

Supplement: Supplementary Figure 6 — Representative frequency histogram of ictal events after PB+BTN treatment at P7, P10, and P12. Frequency histograms of EEG identified ictal events depicted as 1 h slots for the 3 h recording period (top to bottom panel) for both P7 and P10 ligated-control and ligated-treated pups. The pups represent mean seizing rates for the data set depicted in Figure 2. The ictal event distribution graphs represent the lack of seizure clustering during the 3 h recording period for the model. The post-BTN 3rd h recording in a P10 pup also represents the age-specific BTN-induced blunt of PB-subdued seizures in the 3rd h of recording. BTN pharmacokinetics are known to be short (≤30 min; Cleary et al., 2013), but the BTN aggravation persisted for the entire 1 h duration of EEG seizure burden. [file Image6.TIF]
