# Supplementary material for: Age- and sex-dependent susceptibility to phenobarbital-resistant neonatal seizures: role of chloride co-transporters
Source: Front Cell Neurosci. 2015 May 12;9:173. doi: 10.3389/fncel.2015.00173 (PMC4429249; doi:10.3389/fncel.2015.00173)
Supplement: Supplementary file 9 [file Table1.DOCX]

**Supplementary Table 1.** Sample sizes for sham and ligated pups for each age group for EEG and histology experiments.

| **Age** | | | **P7** | **P10** | **P12** | **Sum** |
| --- | --- | --- | --- | --- | --- | --- |
| **EEG** | **Sham** | | 5 | 4 | 3 | 12 |
|  | **Ligated** | **Untreated** | 9 | 8 | 9 | 26 |
|  | **Ligated** | **PB + BTN** | 20 | 16 | 18 | 54 |
|  | **Sum** | | 34 | 28 | 30 | 92 |
| **P18 Histology: CV stain** | | | 15 | 15 | 19 | 49 |
